# The effect of maternal high-risk fertility behavior on child nutritional status in Sub-Saharan Africa: a propensity score-matched analysis

**DOI:** 10.3389/fpubh.2025.1512392

**Published:** 2025-06-04

**Authors:** Beminate Lemma Seifu, Hiwot Atlaye Asebe, Bezawit Melak Fente, Mamaru Melkam, Zufan Alamrie Asmare, Angwach Abrham Asnake, Meklit Melaku Bezie, Yohannes Mekuria Negussie

**Affiliations:** ^1^Department of Public Health, College of Medicine and Health Sciences, Samara University, Samara, Ethiopia; ^2^Department of General Midwifery, School of Midwifery, College of Medicine and Health Sciences, University of Gondar, Gondar, Ethiopia; ^3^Department of Psychiatry, College of Medicine and Health Science, University of Gondar, Gondar, Ethiopia; ^4^Department of Ophthalmology, School of Medicine and Health Science, Debre Tabor University, Debre Tabor, Ethiopia; ^5^Department of Epidemiology and Biostatistics, School of Public Health, College of Medicine and Health Sciences, Wolaita Sodo University, Wolaita Sodo, Ethiopia; ^6^Department of Public Health, Institute of Public Health, College of Medicine and Health Sciences, University of Gondar, Gondar, Ethiopia; ^7^Department of Medicine, Adama General Hospital and Medical College, Adama, Ethiopia

**Keywords:** high-risk fertility behavior, stunting, Sub-Saharan Africa, underweight, wasting, propensity score-matched

## Abstract

**Introduction:**

Maternal high-risk fertility behaviors (HRFBs) represent a multifaceted bio-demographic factor with profound implications for child health. Maternal HRFBs raise the risk of undernutrition in children under the age of five, rendering them more susceptible to stunting and wasting. When estimating the impact of HRFBs on child malnutrition using an observational study such as the Demographic and Health Survey (DHS), it’s important to consider the potential for selection bias and the effect of confounding variables. To address this, we employed propensity score-matched (PSM) analysis to more accurately estimate the true impact of maternal HRFBs on children’s nutritional status.

**Methods:**

Secondary data analysis of 161,179 children under the age of five was conducted based on the 26 Sub-Saharan Africa DHS data (2015–2023). To estimate the maternal HRFBs on children’s nutritional status, We employed propensity score matching (PSM) with a logistic model using the ‘psmatch2 ate’ STATA command to estimate the Average Treatment Effect (ATE) for the population, the treated group (ATT), and the untreated group (ATU). We assessed the quality of matching both statistically and graphically. Additionally, we conducted a sensitivity analysis to test the robustness of the PSM estimates, using the Mantel–Haenszel (MH) test statistic.

**Results:**

More than two-thirds of (57.14, 95%CI: 56.90, 57.39) mothers had a high-risk fertility behavior. In SSA the prevalence of stunting, wasting, and being underweight among under-five children was 30.58% (95%CI: 76.30, 76.72), 6.74% (95%CI: 6.62, 6.86) and 16.70% (95%CI: 16.52, 16.88), respectively. The difference in the Average Treatment Effect (ATE) of maternal HRFBs on stunting, wasting, and underweight was 2.00, 0.30, and 2.19%, respectively. The Average Treatment Effect on the Treated group (ATT) showed a 2.12% increased risk of stunting, a 0.24% decreased risk of wasting, and a 2.68% increased risk of underweight. These estimates remained robust to hidden bias and demonstrated good matching quality.

**Conclusion:**

These results indicate the necessity of public health interventions aimed at enhancing maternal and child health by promoting family planning services, educating young mothers, and providing support to women with high parity.

## Introduction

Childhood malnutrition is a major global public health concern, especially in resource-limited settings. A child’s nutritional status, primarily determined by growth in height and weight, is directly influenced by food intake and the occurrence of infections (1, 2). Malnutrition adversely affects brain development and linear growth in children, leading to both short-term and long-term health consequences, while also diminishing the economic productivity of countries (3, 4).

Globally, an estimated 149 million children under the age of 5 are stunted, 45 million are wasted, and 37 million are overweight or obese (1). In Africa, the prevalence of child malnutrition is 33.2% for stunting, 7.1% for wasting, and 16.3% for being underweight (5). Each year, 6.3 million children under five die worldwide, with almost half of these deaths attributed to undernutrition. Sub-Saharan Africa (SSA) carries one of the highest burdens of undernutrition (6, 7). Evidence from the Millennium Development Goals (MDG) shows that the global rate of underweight children dropped from 25% in 1990 to 15% in 2015 (8). However, this progress was uneven, with almost 90% of underweight children found in South East Asia and SSA. While the number of stunted children decreased in most regions, it rose by about one-third in SSA between 1990 and 2013 (9). Although chronic undernutrition is becoming less common, the number of stunted children under five is rising in SSA due to population growth (10).

Maternal high-risk fertility behaviors (HRFBs) represent a multifaceted bio-demographic factor with profound implications for child health. HRFBs are defined as reproductive behaviors that elevate the risk of adverse health outcomes for both the mother and her offspring. These behaviors include early and late childbearing, high parity, and narrow birth intervals (11, 12). Studies indicate that HRFBs are connected to unfavorable health consequences for mothers, infants, and children, encompassing stillbirth, premature birth, child malnutrition, low birth weight, and higher rates of infant and neonatal mortality (12–15). Additionally, maternal HRFBs increase the risk of undernutrition in children under five, rendering them more susceptible to stunting and wasting (16, 17).

In low-and middle-income countries (LMICs), the impact of HRFBs is notably exacerbated by widespread poverty, inadequate health infrastructure, limited healthcare accessibility, unmet needs for family planning, and early child marriage (13, 18, 19). In SSA, children frequently experience nutritional challenges, evident from the substantial rates of stunting (31.3%) and wasting (8.1%) (17).

The majority of studies conducted in SSA were predominantly observational and relied on traditional regression methodologies to investigate the factors associated with a child’s nutritional status. While HRFBs are established determinants of poor child health, they are closely tied to socioeconomic factors, requiring robust evaluation methods like Propensity Score Matching (PSM) to isolate their effects. To our knowledge, this is the first study in SSA to employ PSM as a method to analyze the actual impact of maternal high-risk fertility behavior on child nutritional status.

## Methods

### Study design, period, sample size, and sampling procedure

The current study utilized data from the Demographic and Health Surveys (DHS) of 26 SSA countries spanning the period from 2015 to 2023. The DHS is a nationally representative survey designed to gather information on key indicators related to population dynamics, nutrition, and health using a community-based cross-sectional study design. A two-stage stratified sampling method was employed to identify participants for the study. In the initial stage, Enumeration Areas (EAs) were randomly chosen based on recent population data, utilizing the housing census as a sampling frame. Subsequently, households were selected in the second stage. For this particular study, focusing on under-five children, the dataset derived from the kid’s records (KR) file was utilized. Additional information on the DHS methodology is available at https://dhsprogram.com/Methodology/index.cfm. In this study, a total weighted sample of 161,179 children under the age of five from 26 SSA countries was considered in the final analysis.

### Study variables

#### Outcome variable

Stunting: defined as the children with height-for-age Z-score (HAZ) < −2SD.

Wasting: defined as the children with weight-for-height Z-score (WHZ) < −2SD.

Underweight: defined as the children with weight-for-age Z-score (WAZ) < −2SD (20).

#### Exposure variable

The exposure variable was maternal high-risk fertility behavior.

High-risk fertility behavior categories were:

1) Children born to mothers under age 18 years.
2) Children born to mothers 35 years and older.
3) Children of birth order 4 or higher.
4) Children born less than 24 months since a preceding birth.

We gave 1 if any single risk factors (mother’s age at birth <18 years of >34 years, birth interval of <24 months, and birth order of greater than 3) were present and 0 otherwise. We used the DHS definition of “Percentage of births in high-risk fertility behavior categories.”

#### Confounding variables

Many maternal pre-intervention characteristics have been included in the model as they ensure a better chance that the PSM assumption holds. Variables that have an effect on HRFB and child nutritional status at the same time but which are not affected by the exposure (HRFB) were included. Variables such as country, residence, maternal age, maternal educational level, maternal employment status, husband/partner’s educational level, household wealth index, sex of household head, media exposure, health insurance coverage, and perceived distance to health facility were considered for matching. Confounding variables that have a significant association with the exposure and outcome variables were considered for matching.

### Propensity score and average treatment effect

In experimental studies, particularly Randomized Controlled Trials (RCTs), study participants are randomly assigned to either the exposure or control group. Randomization helps control both known and unknown confounding factors. In observational studies, unlike in RCT, randomization cannot be employed to allocate study participants to different groups. This inability results in inherent imbalances in observed variables, introducing a bias that can significantly influence the causal effect of the exposure. When confounding variables are measurable, we must make adjustments to address and rectify any imbalances between groups. The balancing score, which is a function of the observed covariates, can be utilized to address the imbalance between the control and exposure groups. According to the balancing score, the observed variables should be unrelated to the assignment of the exposure, whether it’s HRFB or no HRFB.

The propensity score method is commonly used to balance the inequality of the confounding variables in observational studies. Using propensity score matching, the difference in children’s nutritional status between the children whose mothers had HRFB and children whose mothers had no HRFB will be an unbiased estimate of the effect of HRFB after controlling for the observed covariates through propensity scores. A propensity score denotes the probability of a patient receiving an exposure (HRFB) based on all the observed covariates. This score represents the conditional probability of being exposed (HRFB) and consequently falls within the range of 0–1. The likelihood of a child being born from a mother who had HRFB increases with a higher propensity score. In a propensity score analysis, the exposure variable of interest must be dichotomous.

The observed covariates can be grouped into three categories based on their relationships with exposure and outcome: covariates that are only related to the exposure assignment, covariates related to both exposure assignment and outcome (known as confounders), and covariates that are only related to outcome. In this study, only the confounders were included in the propensity score model.

The propensity score for each child is computed based on the identified confounding variables, mitigating the probability of adverse effects for children whose mothers had HRFB. Given that the exposure variable under consideration is dichotomous (maternal HRFB vs. no maternal HRFB), common approaches to generating propensity scores involve logistic regression or discriminant analysis.

The impact of maternal HRFB on children’s nutritional status was estimated using Propensity Score Matching (PSM). This statistical approach is widely used to address the main limitation of establishing causal inference from observational research designs when randomization is not feasible for creating exposed and control groups. The process involves creating matched sets of control and exposed groups consisting of individuals with similar propensity scores. Following the establishment of these matched samples, the impact of maternal HRFB can be evaluated by directly comparing rates of stunting, wasting, and underweight between children whose mothers had HRFB and those whose mothers did not within the matched sample.

The propensity score matching (PSM) approach was employed due to the non-random assignment of maternal HRFB, which can be significantly influenced by observable and non-observable variables. Matching variables were chosen based on their significant association with maternal HRFB and child nutritional status. The study matched children whose mothers had HRFB with children whose mothers did not have HRFB using logit regression (psmatch2 STATA command). Additionally, we used pstest to evaluate the balance of all covariates before and after matching, considering a significance level of 5% or higher as indicative of imbalance.

We endeavor to assess the impact of maternal HRFB on the beneficiaries. Let AiT indicate the nutritional status of children whose mothers were subjected to HRFB (the exposed cohort), and let AiC designate the nutritional status of children whose mothers were not subjected to HRFB. The observed outcome can be written as Ai = (1 − Ti)AiC + TiAiJ, where Ti = 0, 1 denotes exposure assignment (HRFB). The gain from the exposure (HRFB) is (AiT − AiC) and our interest is to estimate the average effect of exposure (maternal HRFB) on the exposed (ATT), E(Ai^T^ − Ai^C^/Ti = 1). This cannot be estimated directly since neither are normally observed as Ai^T^ for Ti = 0 and Ai^C^ for Ti = 1 are not known.

The selection of variables for matching was based solely on their conceptualization before the exposure (HRFB). A rigorous assessment of PSM assumptions, including common support and selection of unobservable factors, was conducted through both graphical and statistical methods. During the analysis, the option of constraining the testing of balancing propensity was contemplated to include solely those children exposed to maternal HRFB exposure whose propensity score for children’s nutritional status aligned within the range of propensity scores for the control group. When utilizing the “*pstest*” command to evaluate covariate balance, we examined several matching methods, including nearest neighbor matching with and without replacement, and radius matching with calipers set at 0.01. The psmatch2 command was utilized to compute the Average Treatment Effect on the Treated (ATT) and Average Treatment Effect (ATE) for the matching method that yielded the highest quality matches. Additionally, the common support option was incorporated to ensure the generation of superior quality matches.

The quality of matching was assessed by computing the standardized bias before and after matching to evaluate the balance of covariates between the treated and control groups. The bias is determined by computing the percentage difference between the sample mean in the exposed and matched control groups, which is then divided by the square root of the average of the sample variances in both groups. In the absence of a definitive standard, it is important to note that there is no established threshold indicating an imbalance in standardized difference. However, it is generally accepted that a variance of less than 10% is indicative of a negligible difference.

A sensitivity analysis was undertaken to assess the robustness of the Propensity Score Matching (PSM) estimates. Given that the outcome variables were dichotomous, the Mantel–Haenszel (MH) test statistic was employed to evaluate whether the PSM estimates were susceptible to hidden bias. The gamma coefficient denotes the extent to which an unobserved confounder or hidden bias influences the allocation of the intervention to the treated and control groups. The gamma value, ranging from 1 to 2 with a 0.05 increment, can be calculated using the mhbounds STATA command.

### Ethical consideration

Permission to get access to the data was obtained from the measure DHS program online request from http://www.dhsprogram.com.website and the data used were publicly available with no personal identifier.

## Results

### Socio-economic and health-related characteristics of the study participants

A total of 161,179 children under the age of five were included from 26 SSA countries. Forty-eight percent of mothers were between 25 and 35 years old, and 41.61% had no formal education. Only one-third (33.41%) of them reside in rich households and the vast majority (78.88%) live in households headed by males. Regarding health insurance coverage, 90.65% are not covered by health insurance.

**Figure 1:**
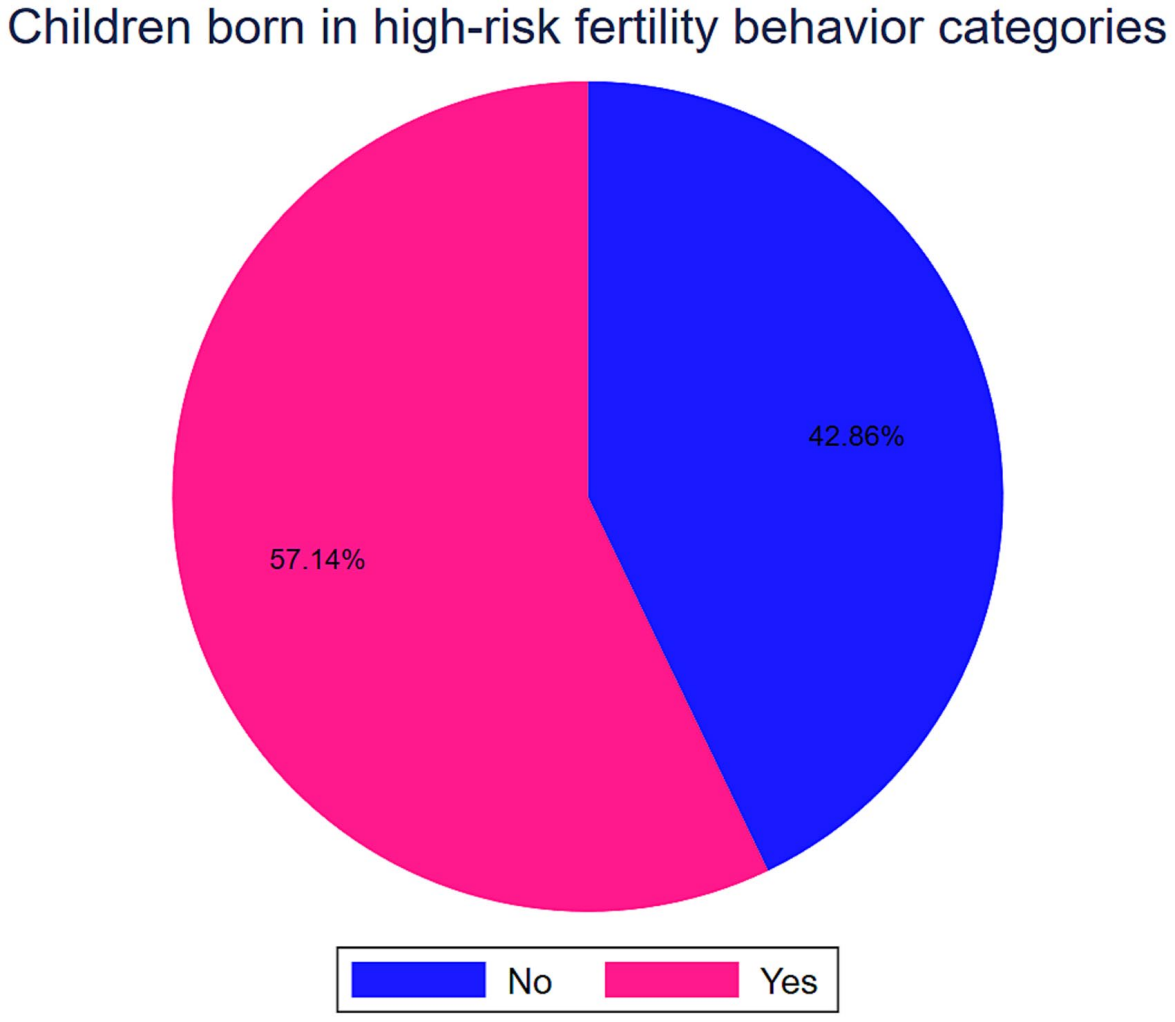
Percentage of births in high-risk fertility behavior categories in SSA.

Of the total 161,179 children under the age of five, 92,104 (57.14, 95%CI: 56.90, 57.39) were born in high-risk fertility behavior categories (Figure 1). In SSA the prevalence of stunting, wasting, and being underweight among under-five children was 30.58% (95%CI: 76.30, 76.72), 6.74% (95%CI: 6.62, 6.86) and 16.70% (95%CI: 16.52, 16.88), respectively (Table 1).

**Table 1:** Socio-economic and health-related characteristics of the study participants.

Variable	Frequency	Percentage
Maternal age		
15–24	44,638	27.69
25–34	77,865	48.31
35–49	38,676	24.00
Maternal educational level		
No formal education	67,074	41.61
Primary	49,312	30.59
Secondary	37,941	23.54
Higher	6,852	4.25
Maternal employment status		
Not employed	65,786	40.85
Employed	95,256	59.15
Husband/partner’s educational level		
No formal education	58,257	40.89
Primary	38,437	26.98
Secondary	34,988	24.56
Higher	10,777	7.56
Household wealth index		
Poor	75,164	46.63
Middle	32,170	19.96
Rich	53,845	33.41
Sex of household head		
Male	127,139	78.88
Female	34,040	21.12
Media exposure		
No	61,850	38.42
Yes	99,148	61.58
Covered by health insurance		
No	116,126	90.65
Yes	11,976	9.35
Distance to a health facility		
Big problem	54,754	38.31
Not a big problem	88,180	61.69
Residence		
Urban	49,563	30.75
Rural	111,616	69.25
High-risk fertility behavior		
No	69,075	42.86
Yes	92,104	57.14
Stunting (160,129)		
No	111,157	69.42
Yes	48,972	30.58
Wasting (160,318)		
No	149,508	93.26
Yes	10,810	6.74
Underweight (160,172)		
No	133,421	83.30
Yes	26,751	16.70

All baseline characteristics such as country, residence, maternal age, maternal educational level, maternal employment status, husband/partner’s educational level, household wealth index, sex of household head, media exposure, health insurance coverage, and perceived distance to health facility had a significant association with maternal high-risk fertility behavior, child stunting, wasting and underweight (Table 2).

**Table 2:** Factors associated with high-risk fertility behavior.

Variables	High-risk fertility behavior		*p*-value	Stunting		*p*-value	Wasting		*p*-value	Underweight		*p*-value
	No	Yes		No	Yes		No	Yes		No	Yes	
Country												
Angola	36.69	63.31	<0.001	62.70	37.30	<0.001	94.97	5.03	<0.001	81.45	18.55	<0.001
Burkina Faso	46.27	53.73		78.80	21.20		90.13	9.87		83.81	16.19	
Benin	45.35	54.65		68.27	31.73		94.82	5.18		83.49	16.51	
Burundi	42.62	57.38		45.49	54.51		94.97	5.03		71.25	28.75	
Cote dIvoire	42.85	57.15		75.98	24.02		91.47	8.53		85.88	14.12	
Cameroon	40.18	59.82		71.86	28.14		96.06	3.94		90.12	9.88	
Ethiopia	38.31	61.69		63.66	36.34		87.91	12.09		75.13	24.87	
Ghana	49.94	50.06		81.87	18.13		94.20	5.80		87.04	12.96	
Gambia	42.68	57.32		81.92	18.08		94.88	5.12		87.83	12.17	
Guinea	40.23	59.77		69.27	30.73		91.40	8.60		84.34	15.66	
Kenya	49.12	50.88		82.07	17.93		92.82	7.18		87.50	12.50	
Liberia	39.22	60.78		67.99	32.01		95.78	4.22		88.24	11.76	
Madagascar	44.15	55.85		61.22	38.78		92.39	7.61		76.63	23.37	
Mali	33.43	66.57		73.45	26.55		90.56	9.44		81.39	18.61	
Malawi	50.47	49.53		64.77	35.23		96.95	3.05		88.45	11.55	
Mozambique	44.40	55.60		66.94	33.06		96.79	3.21		87.06	12.94	
Nigeria	37.83	62.17		63.94	36.06		93.37	6.63		78.79	21.21	
Rwanda	53.94	46.06		66.66	33.34		98.87	1.13		92.49	7.51	
Sierra Leone	47.41	52.59		70.00	30.00		94.44	5.56		86.32	13.68	
Senegal	43.75	56.25		80.58	19.42		90.97	9.03		84.36	15.64	
Chad	26.83	73.17		57.09	42.91		85.83	14.17		67.50	32.50	
Tanzania	45.51	54.49		71.18	28.82		96.38	3.62		88.73	11.27	
Uganda	36.99	63.01		71.59	28.41		96.26	3.74		89.61	10.39	
South Africa	66.14	33.86		75.35	24.65		97.21	2.79		94.51	5.49	
Zambia	43.16	56.84		65.26	34.74		95.93	4.07		88.12	11.88	
Zimbabwe	58.57	41.43		74.75	25.25		96.52	3.48		92.53	7.47	
Maternal age												
15–24	62.79	37.21	<0.001	68.29	31.71	<0.001	93.12	6.88	0.416	83.37	16.63	<0.05
25–34	48.85	51.15		70.12	29.88		93.30	6.70		83.50	16.50	
35–49	7.78	92.22		69.30	30.70		93.32	6.68		82.81	17.19	
Maternal educational level												
No formal education	30.35	69.65	<0.001	64.39	35.61	<0.001	90.56	9.44	<0.001	77.30	22.70	<0.001
Primary	40.74	59.26		67.33	32.67		95.07	4.93		85.26	14.74	
Secondary	62.45	37.55		77.33	22.67		95.18	4.82		89.42	10.58	
Higher	72.01	27.99		89.91	10.09		96.02	3.98		94.08	5.92	
Maternal employment status												
Not employed	44.97	55.03	<0.001	70.12	29.88	<0.001	91.87	8.13	<0.001	82.14	17.86	<0.001
Employed	41.41	58.59		68.96	31.04		94.23	5.77		84.12	15.88	
Husband/partner’s educational level												
No formal education	32.22	67.78	<0.001	65.28	34.72	<0.001	90.47	9.53	<0.001	77.58	22.42	<0.001
Primary	38.73	61.27		66.68	33.32		94.80	5.20		84.27	15.73	
Secondary	52.60	47.40		74.41	25.59		94.98	5.02		88.17	11.83	
Higher	63.55	36.45		85.72	14.28		95.07	4.93		91.97	8.03	
Household wealth index												
Poor	36.32	63.68	<0.001	63.75	36.25	<0.001	92.25	7.75	<0.001	79.56	20.44	<0.001
Middle	41.90	58.10		68.97	31.03		93.88	6.12		83.92	16.08	
Rich	52.55	47.45		77.60	22.40		94.30	5.70		88.14	11.86	
Sex of household head												
Male	41.74	58.26	<0.001	69.12	30.88	<0.001	93.19	6.81	<0.05	83.02	16.98	<0.001
Female	47.04	52.96		70.51	29.49		93.53	6.47		84.34	15.66	
Media exposure												
No	35.46	64.54	<0.001	62.13	37.87	<0.001	91.36	8.64	<0.001	77.87	22.13	<0.001
Yes	47.49	52.51		73.98	26.02		94.45	5.55		86.71	13.29	
Covered by Health insurance												
No	42.33	57.67	<0.001	67.55	32.45	<0.001	93.80	6.20	<0.001	83.44	16.56	<0.001
Yes	51.40	48.60		74.15	25.85		95.77	4.23		88.32	11.68	
Distance to a health facility												
Big problem	39.08	60.92	<0.001	66.10	33.90	<0.001	93.09	6.91	<0.001	82.11	17.89	<0.001
Not a big problem	46.37	53.63		71.65	28.35		94.23	5.77		85.40	14.60	
Residence												
Urban	51.31	48.69	<0.001	77.16	22.84	<0.001	94.16	5.84	<0.001	87.95	12.05	<0.001
Rural	39.10	60.90		65.99	34.01		92.86	7.14		81.24	18.76	

### Estimation of propensity scores

The logit model was employed for estimating the propensity score of maternal high-risk fertility behavior in the study population (Table 3). The average propensity score was 0.583, and there was minimal variability (Sd = 0.232) between the intervention group (high-risk fertility behavior) and the control group (no high-risk fertility behavior). This value represents the average estimated probability of being exposed (being in the HRF group) based on observed covariates. In other words, it indicates how likely an individual is to be treated given their characteristics.

**Table 3:** Logit regression analysis of factors associated with high-risk fertility behavior in SSA.

Variables	High-risk fertility behavior	
	Coefficient	*p*-value
Country	0.010	0.000
Residence	0.117	0.000
Maternal age	1.384	0.000
Maternal educational level	−0.380	0.000
Maternal employment status	0.072	0.000
Husband/partner’s educational level	−0.084	0.000
Household wealth index	−0.180	0.000
Sex of household head	−0.154	0.000
Media exposure	−0.048	0.002
Covered by health insurance	−0.421	0.000
Distance of health facility	−0.069	0.000
Constant	−1.588	0.000

### The impact of maternal high-risk fertility behavior on under-five child nutritional status

We estimated the impact of HRFB on child nutritional status by the estimated difference between the treated groups (maternal HRFB) and the matched control groups (no maternal HRFB). The Propensity Score Matching (PSM) analysis calculates the effect of the HRFB exposure while accounting for background variables associated with HRFB and child nutritional status.

Propensity Score Matching is a technique used to estimate the ATT by creating a comparison group similar to the exposed group based on observed covariates. This helps assess the exposure’s impact on those who received it. PSM can also be adapted to estimate the ATE, which considers the exposure’s effect on the entire population, including both treated and untreated groups.

A radius matching approach with a 0.01 caliper width provided the highest quality of matching and was employed to estimate the average treatment effect of HRFB on the overall population, the average treatment effect on those who are exposed, and the average effect on those who did not exposed.

The unmatched estimate showed that births from women who had high-risk fertility behavior had a 5.63, 0.59, and 3.86% increased risk of stunting, wasting, and underweight among under-five children, respectively.

The ATE of high-risk fertility behavior on stunting, wasting, and underweight was 2.00, 0.30, and 2.19, respectively, showing that maternal high-risk fertility led to 2.00, 0.30, and 2.19% increments in childhood stunting, wasting and underweight. The ATT was 2.12, and 2.68% increased risk of stunting and underweight, respectively. Similarly, the contrast in estimated average treatment effects (HRFB) in untreated groups between the treated and control groups was 1.72% for stunting. Conversely, the average treatment effects in untreated groups were 1 and 1.68%, indicating that the control groups experienced a decrease in the risk of wasting and underweight compared to the exposed group (Table 4).

**Table 4:** A propensity score-matched analysis of the impact of maternal high-risk fertility on child stunting, wasting, and underweight.

Impact of high-risk fertility behavior on child nutritional status	Treated (%)	Control (%)	Difference (%)	SE	*p*-value	t-statistics
Impact of HRFB on stunting						
Unmatched	33.99	28.43	5.56	0.003		16.65
ATT	33.99	31.87	2.12	0.001	0.000	0.31
ATU	28.43	30.15	1.72			
ATE			2			
Impact of HRFB on wasting						
Unmatched	6.51	5.54	0.97	0.001		6.70
ATT	6.51	6.75	−0.24	0.006	0.015	−0.41
ATU	5.54	6.54	1			
ATE			0.30			
Impact of HRFB on underweight						
Unmatched	17.88	13.84	4.04	0.27		18.16
ATT	17.88	15.20	2.68	0.86	0.000	3.11
ATU	13.84	15.52	1.68			
ATE			2.19			

### Quality of matching

Since we do not condition on all covariates but on the propensity score, we checked if the matching procedure can balance the distribution of the relevant variables in both the control and exposure groups. The basic idea of all approaches is to compare the situation before and after matching and check if there remain any differences after conditioning on the propensity score.

The pseudo-R2 indicates how well the regressors explain the participation probability. After matching there should be no systematic differences in the distribution of covariates between both groups and therefore, the pseudo-R2 should be fairly low.

For the Unmatched sample, the maximum absolute bias (B) is 115.5% which is quite high, and the maximum absolute % bias reduction (R) is 1.53. For the Matched sample, Maximum absolute bias (B) is reduced to 12.0, 10.9, and 10.3% and R is 1.05, 0.98, and 1.06 indicating a significant improvement in the balance of the covariates after matching (Tables 5–7).

**Table 5:** Performance of the propensity score matching for stunting: quality measurements.

Sample	Ps R2	LR chi2	*p* > chi2	Mean Bias	Med Bias	B	R	%Var
Unmatched	0.180	20,929.45	0.000	30.9	21.9	115.5*	1.53	100
Matched	0.003	635.85	0.000	3.0	3.0	12.0	1.05	75

*p-value <0.05.

**Table 6:** Performance of the propensity score matching for wasting: quality measurements.

Sample	Ps R2	LR chi2	*p* > chi2	Mean Bias	Med Bias	B	R	% Var
Unmatched	0.180	20,948.89	0.000	30.9	21.9	115.5*	1.53	100
Matched	0.002	522.80	0.000	2.9	2.5	10.9	0.98	75

*p-value <0.05.

**Table 7:** Performance of the propensity score matching for wasting: quality measurements.

Sample	Ps R2	LR chi2	*p* > chi2	Mean Bias	Med Bias	B	R	% Var
Unmatched	0.180	20,936.30	0.000	30.9	21.9	115.5*	1.53	100
Matched	0.002	468.07	0.000	2.5	2.6	10.3	1.06	63

*p-value <0.05.

For the Unmatched sample, the maximum absolute bias (B) is 115.5% which is quite high, and the maximum absolute % bias reduction (R) is 1.53. For the Matched sample, B is reduced to 12.0% and R is 1.05, indicating a significant improvement in the balance of the covariates after matching.

It appears that the matching process has significantly reduced the Mean Bias, Median absolute Bias, and B from the ‘Unmatched’ to the ‘Matched’ groups, indicating a successful matching process.

### Common support

Implementing the common support condition ensures that any combination of characteristics observed in the exposed group can also be observed in the control group. The common support is important for propensity score matching because it represents the range of scores for which it is possible to find a match between an exposed and a controlled individual. If there is no overlap in the propensity score distribution, then it would not be possible to find a match and the analysis would not be valid.

When we fitted the propensity-matched analysis of the impact of the HRFB, about 1 observation was dropped due to the common support option for stunting, wasting, and underweight, respectively (Table 8).

**Table 8:** Common support.

Impact of high-risk fertility behavior on child nutritional status	Off support	On support	Total
Impact of HRFB on stunting			
No	1	46,789	46,790
Yes	0	65,334	65,334
Total	1	112,123	112,124
Impact of HRFB on wasting			
No	1	46,831	46,832
Yes	0	65,444	65,444
Total	1	112,275	112,276
Impact of HRFB on underweight			
No	1	46,796	46,797
Yes	0	65,338	65,338
Total	1	112,134	112,135

**Figure 2:**
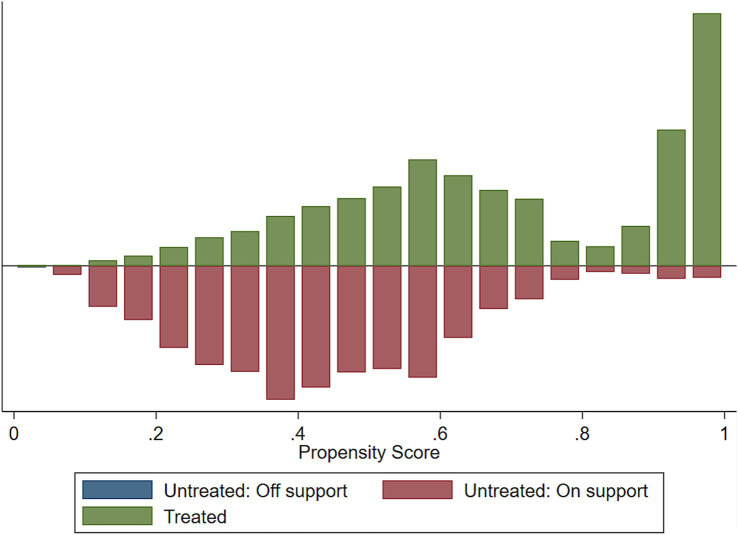
Histogram to show common support.

**Figure 3:**
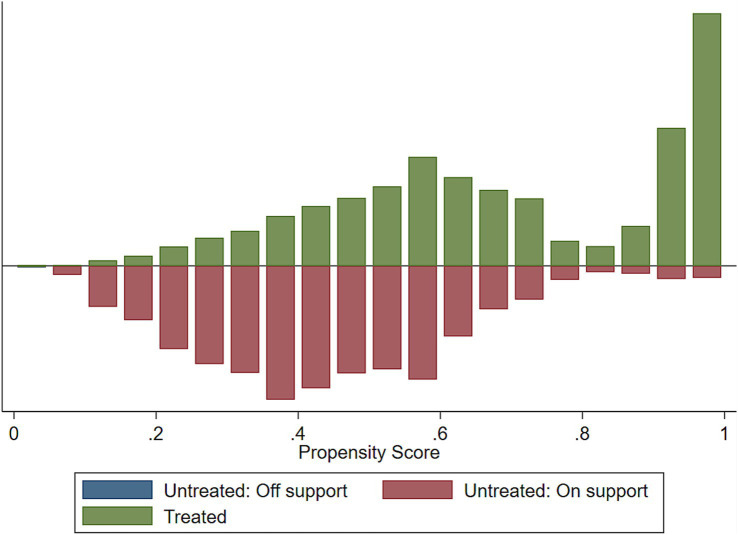
Histogram to show common support.

**Figure 4:**
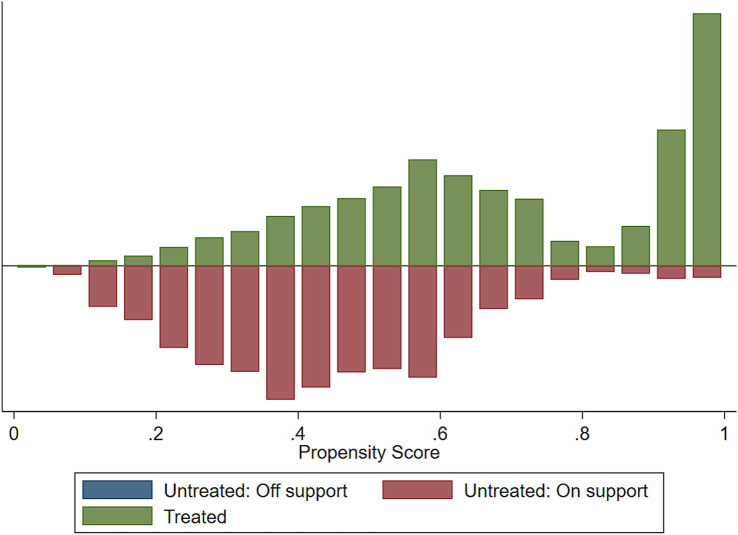
Histogram to show common support.

We assessed the common support assumptions graphically and statistically, and the assumption was fulfilled (Figures 2–4). Observations in the intervention and control groups with propensity score outside the region of common support were not included in the analysis.

### Sensitivity analysis

In the presence of unobserved variables exerting simultaneous influence on both the assignment into exposure and the outcome variable, the emergence of ‘hidden bias’ becomes a concern. It is imperative to recognize that matching estimators lack robustness in addressing this form of bias. Given the unfeasibility of quantifying selection bias magnitude using non-experimental data, we confront this challenge employing the bounding approach proposed by Rosenbaum. In all of the analyses, in a study free of bias, that is, where Ґ = 1, the Q_MH_ statistic in this case provides strong evidence that HRFB causes child malnutrition. The upper bound on the significance level for Ґ = 1.05, 1.1, 1.15…2 was significant. The significance of these results suggests that our study is robust to hidden bias, meaning that even if there were unobserved confounding variables, they would not significantly alter our findings (Tables 9–11).

**Table 9:** Sensitivity analysis using Mantel–Haenszel bounds for stunting.

Gamma (Γ)	Test statistics		Significance level	
	Over-estimation (Q_mh+)	Under-estimation (Q_mh−)	Over-estimation (p_mh+)	Under-estimation (p_mh−)
1	0.186407	0.186407	0.426063	0.426063
1.05	1.40009	0.987006	0.080744	0.16182
1.1	2.55759	2.14437	0.00527	0.016002
1.15	3.66414	3.25071	0.000124	0.000576
1.2	4.72426	4.31055	<0.0000	<0.0000
1.25	5.74189	5.32784	<0.0000	<0.0000
1.3	6.7205	6.30606	<0.0000	<0.0000
1.35	7.66313	7.24827	<0.0000	<0.0000
1.4	8.57252	8.15719	<0.0000	<0.0000
1.45	9.45106	9.03523	<0.0000	<0.0000
1.5	10.3009	9.88458	<0.0000	<0.0000
1.55	11.1241	10.7072	<0.0000	<0.0000
1.6	11.9222	11.5047	<0.0000	<0.0000
1.65	12.697	12.2789	<0.0000	<0.0000
1.7	13.4498	13.0311	<0.0000	<0.0000
1.75	14.1819	13.7626	<0.0000	<0.0000
1.8	14.8946	14.4747	<0.0000	<0.0000
1.85	15.589	15.1684	<0.0000	<0.0000
1.9	16.266	15.8448	<0.0000	<0.0000
1.95	16.9265	16.5047	<0.0000	<0.0000
2.0	17.5715	17.149	<0.0000	<0.0000

**Table 10:** Sensitivity analysis using Mantel–Haenszel bounds for wasting.

Gamma (Γ)	Test statistics		Significance level	
	Over-estimation (Q_mh+)	Under-estimation (Q_mh−)	Over-estimation (p_mh+)	Under-estimation (p_mh−)
1	0.36107	0.36107	0.359023	0.359023
1.05	0.209026	1.00695	0.417214	0.15698
1.1	0.824748	1.62322	0.204757	0.052271
1.15	1.41344	2.21277	0.078764	0.013457
1.2	1.97761	2.77807	0.023986	0.002734
1.25	2.51944	3.32127	0.005877	0.000448
1.3	3.04084	3.84424	0.00118	<0.0006
1.35	3.54348	4.34864	<0.0000	<0.0000
1.4	4.02882	4.83591	<0.0000	<0.0000
1.45	4.49819	5.30735	<0.0000	<0.0000
1.5	4.95275	5.7641	<0.0000	<0.0000
1.55	5.39353	6.20719	<0.0000	<0.0000
1.6	5.82147	6.63755	<0.0000	<0.0000
1.65	6.23743	7.05601	<0.0000	<0.0000
1.7	6.64215	7.46333	<0.0000	<0.0000
1.75	7.03634	7.86018	<0.0000	<0.0000
1.8	7.42063	8.2472	<0.0000	<0.0000
1.85	7.79558	8.62494	<0.0000	<0.0000
1.9	8.16173	8.99394	<0.0000	<0.0000
1.95	8.51956	9.35466	<0.0000	<0.0000
2.0	8.8695	9.70755	<0.0000	<0.0000

**Table 11:** Sensitivity analysis using Mantel–Haenszel bounds for underweight.

Gamma (Γ)	Test statistics		Significance level	
	Over-estimation (Q_mh+)	Under-estimation (Q_mh−)	Over-estimation (p_mh+)	Under-estimation (p_mh−)
1	1.51486	1.51486	0.064903	0.064903
1.05	0.523222	2.5072	0.30041	0.006085
1.1	0.372916	3.45421	0.354605	0.000276
1.15	1.27634	4.36025	0.100917	<0.0000
1.2	2.14169	5.22903	0.016109	<0.0000
1.25	2.97231	6.06379	0.001478	<0.0000
1.3	3.7711	6.86736	0.000081	<0.0000
1.35	4.54063	7.64224	<0.0000	<0.0000
1.4	5.28318	8.39063	<0.0000	<0.0000
1.45	6.00076	9.11449	<0.0000	<0.0000
1.5	6.69516	9.81557	<0.0000	<0.0000
1.55	7.36801	10.4954	<0.0000	<0.0000
1.6	8.02075	11.1555	<0.0000	<0.0000
1.65	8.6547	11.7971	<0.0000	<0.0000
1.7	9.27102	12.4213	<0.0000	<0.0000
1.75	9.87082	13.0292	<0.0000	<0.0000
1.8	10.4551	13.6218	<0.0000	<0.0000
1.85	11.0246	14.1999	<0.0000	<0.0000
1.9	11.5804	14.7643	<0.0000	<0.0000
1.95	12.123	15.3158	<0.0000	<0.0000
2.0	12.6533	15.855	<0.0000	<0.0000

## Discussion

This research endeavors to examine the causal impact of HRFB on child nutritional status employing PSM analysis. This method represents one of the most effective means of evaluating the influence of a specific intervention in observational studies by establishing a suitable comparison group in the absence of randomization. The analysis conducted by previous researchers indicates that HRFB is a significant predictor of child undernutrition. Furthermore, they have documented a notable association between HRFB and the nutritional status of children within Sub-Saharan African (SSA) nations (17, 19). However, we estimated the actual impact of HRFB on under-five children stunting, wasting, and underweight.

The present study revealed a significant and positive impact of HRFB on child malnutrition after matching treated and untreated children on all included observable characteristics. In the PSM analysis, we found the ATEs of HRFB on child stunting, wasting and underweight were 0.29, 0.58, and 2.19%, respectively. The ATT was 0.42, 0.46, and 2.42% increased risk of stunting, wasting and underweight, respectively. This positive causal impact of HRFB on child stunting, wasting, and underweight can be attributed to the fact that Maternal Age, Early or late maternal age at childbirth may affect child nutrition due to inadequate care or resources. Birth Intervals; Short intervals between pregnancies can affect maternal health and child nutrition. High Parity; having many children might strain resources and affect a child’s well-being. Limited resources may lead to inadequate food availability, affecting children’s nutritional intake. Families may struggle to afford healthcare services, vaccinations, and preventive measures for their children (21, 22). When mothers give birth at a very young age (typically below 18 years), several risk factors come into play: Young mothers may not have fully developed physically, affecting their ability to provide adequate care and nutrition to their children (23). Adolescent mothers often live in poor conditions, lack financial resources, experience high stress, face family instability, and have limited educational opportunities, leading to inadequate parent–child interactions and diminished infant development (24). They may face challenges in seeking social support or guidance during pregnancy and early motherhood. These all have an impact on Child Nutrition due to Children born to very young mothers may be at higher risk of malnutrition due to inadequate breastfeeding practices, poor dietary choices, and limited knowledge about child nutrition (25).

Children conceived after shorter intervals are more likely to experience undernutrition (26). Short intervals might not give mothers enough time to recover from pregnancy before the next one. Pregnancy and breastfeeding can deplete nutrient stores, particularly folate. Insufficient time between pregnancies could affect the health of both the mother and the baby (27).

The prevalence of HRFB among mothers in SSA is concerning, with approximately 76.52% exhibiting these behaviors. HRFB can include early or late maternal age at delivery, short birth intervals, and high parity. These behaviors can affect maternal health, child health, and overall family well-being. Given there is a strong relationship between HRFB and child undernutrition. Children born to mothers with HRFB are at higher risk of stunting, wasting and underweight. Addressing HRFB can contribute to better child growth and development. Health education is essential to raise awareness about the risks associated with HRFB. Women need information on family planning, optimal birth spacing, and the importance of maternal health. Encouraging contraceptive use can help prevent unintended pregnancies. Access to family planning services should be improved across SSA. Policies against child marriage are vital to reducing early pregnancies.

## Conclusion

The study’s findings demonstrate a significant and positive causal relationship between HRFB and child undernutrition in Sub-Saharan Africa. These results indicate the necessity of public health interventions aimed at enhancing maternal and child health by promoting family planning services, educating young mothers, and providing support to women with high parity. It is imperative to advocate for policies that prioritize the health of mothers and children, with a specific focus on nutrition. Collaboration between governments and stakeholders is crucial in striving toward the achievement of the Global Nutrition targets. These targets seek to realize a 40% reduction in the prevalence of stunted children under the age of five and to sustain childhood wasting below 5% by the year 2025.

## Strengths and limitations of the study

While this study provides valuable insights into the actual impact of HRFB on child malnutrition, it is essential to interpret the results in consideration of the following limitations. The matching was conducted using only observed variables, which could result in residual confounding due to unobserved variables. Although the Demographic and Health Survey (DHS) is a cross-sectional survey prone to social desirability and recall bias, it has some important advantages. First, it is based on nationally representative DHS data with a remarkable response rate. Second, the DHS uses a standardized questionnaire for data collecting, which ensures uniformity across all 26 countries. Furthermore, this study employs the PSM method to adjust for relevant confounders in estimating the causal relationship between HRFB and child malnutrition.

## Data Availability

The original contributions presented in the study are included in the article/supplementary material, further inquiries can be directed to the corresponding author.
